# Does lumbar spinal degeneration begin with the anterior structures? A study of the observed epidemiology in a community-based population

**DOI:** 10.1186/1471-2474-12-202

**Published:** 2011-09-13

**Authors:** Pradeep Suri, Asako Miyakoshi, David J Hunter, Jeffrey G Jarvik, James Rainville, Ali Guermazi, Ling Li, Jeffrey N Katz

**Affiliations:** 1 Division of PM&R, VA Boston Healthcare System, Boston, USA; 2 Department of Physical Medicine and Rehabilitation, Harvard Medical School, Boston, USA; 3 Department of Physical Medicine and Rehabilitation, Spaulding Rehabilitation Hospital, Boston, USA; 4 Division of Research, New England Baptist Hospital, Boston, USA; 5 Departments of Radiology, Neurological Surgery and Health Services, University of Washington Medical Center, Seattle, USA; 6 Northern Clinical School, The University of Sydney, Sydney, Australia; 7 Department of Radiology, Boston University School of Medicine, Boston, USA; 8 Division of Rheumatology, Immunology and Allergy, Department of Medicine and Department of Orthopedic Surgery, Brigham and Women's Hospital, Harvard Medical School, Boston, USA

**Keywords:** degenerative cascade, degeneration, disk, facet joint, arthritis, lumbar, spine

## Abstract

**Background-:**

Prior studies that have concluded that disk degeneration uniformly precedes facet degeneration have been based on convenience samples of individuals with low back pain. We conducted a study to examine whether the view that spinal degeneration begins with the anterior spinal structures is supported by epidemiologic observations of degeneration in a community-based population.

**Methods-:**

361 participants from the Framingham Heart Study were included in this study. The prevalences of anterior vertebral structure degeneration (disk height loss) and posterior vertebral structure degeneration (facet joint osteoarthritis) were characterized by CT imaging. The cohort was divided into the structural subgroups of participants with 1) no degeneration, 2) isolated anterior degeneration (without posterior degeneration), 3) combined anterior and posterior degeneration, and 4) isolated posterior degeneration (without anterior structure degeneration). We determined the prevalence of each degeneration pattern by age group < 45, 45-54, 55-64, ≥65. In multivariate analyses we examined the association between disk height loss and the response variable of facet joint osteoarthritis, while adjusting for age, sex, BMI, and smoking.

**Results-:**

As the prevalence of the no degeneration and isolated anterior degeneration patterns decreased with increasing age group, the prevalence of the combined anterior/posterior degeneration pattern increased. 22% of individuals demonstrated isolated posterior degeneration, without an increase in prevalence by age group. Isolated posterior degeneration was most common at the L5-S1 and L4-L5 spinal levels. In multivariate analyses, disk height loss was independently associated with facet joint osteoarthritis, as were increased age (years), female sex, and increased BMI (kg/m^2^), but not smoking.

**Conclusions-:**

The observed epidemiology of lumbar spinal degeneration in the community-based population is consistent with an ordered progression beginning in the anterior structures, for the majority of individuals. However, some individuals demonstrate atypical patterns of degeneration, beginning in the posterior joints. Increased age and BMI, and female sex may be related to the occurrence of isolated posterior degeneration in these individuals.

## Background

The importance of spine stability is a central paradigm in spine care. Maintenance of spine stability, through decreasing excessive or abnormal spinal movement, is the rationale for many commonly used treatments ranging from 'lumbar stabilization' rehabilitation to spinal fusion surgery [1]. The spinal degenerative cascade is an important theory related to the concept of spine stability, and was originally popularized by Kirkaldy-Willis. Kirkaldy-Willis described a 'cascade' of degenerative changes affecting the three joint complex comprised of the intervertebral disk anteriorly and the lumbar zygapophyseal ('facet') joints posteriorly. This degenerative cascade consists of three sequential clinical stages: 1) dysfunction, 2) instability, and 3) stabilization [2]. Kirkaldy-Willis described a mutual interrelationship of the intervertebral disk and facet joints, and pointed out that precipitating events in degeneration could begin not only with the intervertebral disk, but also with the facet joints [2,3].

Over time, however, the sequence of spinal degeneration has often been viewed from a more limited standpoint, with the perspective that anterior structure changes affecting the intervertebral disk largely precede- and lead to- posterior structure changes affecting the facet joints [4]. The notion that degeneration begins with the intervertebral disk is described in textbooks of spine care [5-7] and has been supported by some research studies [8-12]. Vernon -Roberts conducted a landmark study of fewer than 100 cadaveric specimens that found that degenerative disk changes were always accompanied by facet joint degenerative changes [9]. This study concluded that disk degeneration was the primary event leading to degenerative spondylosis. In an imaging study of 68 subjects with LBP, Butler et al. also found that facet joint degeneration did not occur in the absence of disk degeneration, but disk degeneration frequently occurred without facet joint degeneration [11]. Butler concluded that disks degenerate before facets. These conclusions were further supported by a recent cross-sectional MRI study of individuals with LBP [12]. Some authors, however, have questioned the view that disk degeneration necessarily precedes facet degeneration [7,13-15]. A prior report notes that facet arthrosis on MRI precedes disk degeneration in 20% of men [8]. Furthermore, in a large study of skeletal specimens (n = 647), Eubanks et al. found that facet joint osteoarthritis often preceded changes of disk degeneration in younger individuals [13].

Many prior studies of the degenerative cascade that have concluded that disk degeneration uniformly precedes facet degeneration have been based on convenience samples of individuals with LBP [8,9,11,12]. Given the prevailing notion among clinicians that much LBP originates from the disk [16], recruitment from spine clinics therefore presents a probable selection bias in prior samples. No studies have examined a large, community-based sample that is unselected for LBP. Furthermore, no studies examining the interrelationships between anterior and posterior structure degeneration have used multivariate analyses to adjust for important demographic and anthropometric factors thought to be related to spinal degeneration. We conducted an epidemiologic study of patterns of degeneration in the community-based population of the Framingham Heart Study. The aims of the present study were: 1) to determine the prevalence of different patterns of anterior and posterior spinal structure degeneration in the community-based population, and 2) to examine whether the observed epidemiology is consistent with the view that degeneration always begins with the intervertebral disks, and 3) to determine the independent relationship between anterior structure and posterior structure degeneration, while adjusting for important demographic and anthropometric factors.

## Methods

### Study Sample

We conducted an ancillary study to the Framingham Heart Study. The Framingham Heart Study began in 1948 as a longitudinal population-based cohort study of the causes of heart disease. 5209 men and women living in Framingham, Massachusetts were enrolled in the Original study cohort. Beginning in 1971, 5,124 children of the Original cohort members and their spouses were enrolled as the Offspring cohort [17]. In 2002, 4095 children of the Offspring cohort were enrolled in the Third Generation cohort [18]. 3529 participants in the Offspring and Third Generation cohorts underwent abdominal and chest multi-detector computed tomography (MDCT) scanner to assess coronary and aortic calcification as well as lumbar spine degeneration. The recruitment and conduct of CT scanning have been previously reported [19,20]. 435 participants from the Third Generation and Offspring cohorts participating in the MDCT study were selected randomly for inclusion in this substudy. The Institutional Review Board of New England Baptist Hospital approved this ancillary study.

### CT Evaluation of Spinal Degeneration

Study participants were imaged with a MDCT scanner using methods that have been described previously [19]. Evaluation of spinal degeneration was performed using eFilm Workstation (Version 2.0.0) software. A reading protocol for evaluation of spinal degeneration using established grading criteria was developed. CT assessment of degeneration was performed by a board-certified, fellowship-trained physiatrist researcher specializing in spine care (PS), who was blinded to clinical information. An atlas of the grading criteria for each degenerative parameter was created and used throughout the reading process. The physiatrist reader who performed all CT assessments was calibrated to the standard of a musculoskeletal radiologist with extensive experience with research studies of spinal imaging. Inter-observer reliability was calculated by comparison to CTs that were also read by the musculoskeletal radiologist.

### Grading of Disk Height Loss (DHL)

DHL was graded using a system developed for research by Videman et al., and has been widely used in prior studies [21-23]. Using sagittal CT reformatting and bone algorithm, the midsagittal plane was identified at each spinal level by alignment of the mid-anterior vertebral margin with the spinous process, or at levels where spinous process alignment was notably asymmetric, by alignment with the base of the spinous process. DHL was measured in millimeters in the midsagittal plane at the midpoint of the anteroposterior diameter of the disk. DHL was graded as '0' (normal; disk height greater than disk space immediately superior), '1' (mild; disk height equal to disk space immediately superior), '2' (moderate; disk height narrowing as compared to disk space immediately superior), and '3' (severe; endplates almost in contact). The L5-S1 interspace was graded using a 0-3 grade scale based on reader experience, due to the fact that there is greater variability of L5-S1 disk height in relation to other spinal levels, independent of degeneration [24]. This system of classification also necessitates a determination based on reader experience in situations where a superior (reference) disk space appears narrowed.

### Grading of Facet Joint Osteoarthritis (FJ OA)

FJ OA was graded using criteria designed for research purposes that have been used in multiple studies [25,26], and are based on earlier criteria by Pathria et al [27]. and Weishaupt et al [28]. This system grades facet joint OA as grade I (normal), grade II (mild), grade III (moderate), and grade IV (severe) according to the individual subcategories of joint space narrowing (JSN), osteophytes, articular process hypertrophy, sclerosis, subarticular erosions, subchondral cysts, and vacuum phenomenon. FJ OA evaluation was performed using axial images and bone algorithm, with corroboration using sagittal and coronal reformats.

### Reliability of CT assessment

Inter-observer reliability was calculated between the physiatrist and the radiologist at the start of the reading process. All CT scans were interpreted for each parameter at spinal levels L2-L3, L3-L4, L4-L5, and L5-S1. Recalibration of physiatrist to radiologist was performed at multiple points during the reading process, either by direct interaction with the radiologist or by the review of images previously interpreted by the radiologist. To evaluate for reader-drift, we planned in advance to periodically reassess reliability at regular intervals. Inter-observer reliability using the weighted κ statistic varied between 0.70 and 0.84 for DHL (n = 50), and between 0.68 and 0.84 for FJ OA (n = 46).

### Statistical analysis

Analyses focused on the individual participant, rather than on the individual spinal level. This analytic approach addresses the fact that changes at one level are thought to lead to multilevel changes over time [29], and accounts for the fact that different spinal levels in the same individual are not independent of one another. Individuals with spondylolysis, lumbosacral transition levels, and prior spine surgery were excluded in order to eliminate the contributions of these factors, which may alter 'normal' spine biomechanics. Based on the two-column model of Holdsworth [30], we defined anterior structure degeneration as the presence of at least moderate DHL at one of the L2-S1 spinal levels, and posterior structure degeneration as the presence of at least moderate FJ OA at one of the L2-S1 spinal levels. The prevalence of anterior and posterior vertebral structure degeneration was characterized as frequencies and proportions per individual. Individuals were categorized into one of four distinct patterns of spinal degeneration: 1) no degeneration, 2) isolated anterior degeneration (without posterior degeneration), 3) combined anterior and posterior degeneration, and 4) isolated posterior degeneration (without anterior structure degeneration). We determined the prevalence of each degeneration pattern by decade of age. We then used multivariate logistic regression to examine associations between the primary predictor variable of any anterior structure degeneration (either isolated or combined), and the response variable of any posterior structure degeneration (either isolated or combined), while adjusting for age, sex, BMI, and tobacco smoking in the past year. For each stage of the analytic plan, we conducted secondary analyses using an alternate (less conservative) definition for anterior structure degeneration, as defined by the presence of any DHL at one of the L2-S1 spinal levels. All statistical analyses were performed using SAS software, (SAS Institute Inc, Cary, North Carolina, release 9.2).

## Results

435 individuals were evaluated for DHL and FJ OA by CT scan. 45.5% of individuals were female, and the mean age of the sample was 58.0 ±13.1 years. 6.0% of individuals had a lumbosacral transitional level, 8.3% had spondylolysis, and 3.2% had prior lumbar spinal surgery; these individuals (n = 74) were excluded from subsequent analyses, leaving 361 participants in the analysis. Individuals who were excluded were older than those who were not excluded (63.0 vs. 57.0; p = 0.0004), but were not materially different with respect to sex and BMI (data not shown).

**Figure 1:**
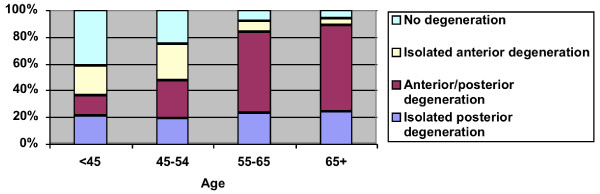
Prevalence of Patterns of Spinal Degeneration by Age Group (Definition 1)*. *Anterior degeneration: at least moderate disk height loss at L2-S1; posterior degeneration: at least moderate facet joint osteoarthritis at L2-S1.

The study sample is described in Table 1. The mean age of the cohort was 57.0 ±13.0 years, 46.5% of individuals were female, and the mean BMI was 28.0 ± 5.1. Table 2 presents the prevalence of the four patterns of degeneration by age group. A graphical display of this information (Figure 1) shows that as the prevalence of the no degeneration pattern and the isolated anterior degeneration pattern decreased with increasing age group, the prevalence of the combined anterior/posterior degeneration pattern increased. Interestingly, the prevalence of isolated posterior degeneration did not appear to substantially increase or decrease with increasing age group, and was prevalent in between 19-24% of individuals. Where isolated posterior degeneration was present, the L2-L3 level was involved in 28% of individuals, L3-L4 in 35% of individuals, L4-L5 in 63% of individuals, and L5-S1 in 66% of individuals.

**Table 1:** Characteristics of the Study Sample (n = 361)

	Mean (SD) or N (%)
**Demographics**	
Age	57.0 (13.0)
Female sex	168 (46.5%)
**Anthropometric features**	
BMI	28.0 (5.1)
**Prevalence of degeneration**	
Moderate Disk Height Loss*	215 (60.6%)
Moderate Facet Joint Osteoarthritis*	241 (66.8%)

*at one of the L2-S1 spinal levels

**Table 2:** Prevalence of Patterns of Spinal Degeneration by Age Group (Definition 1)*

	Age < 45 n = 80	Age 45-54 n = 80	Age 55-64 n = 92	Age 65+ n = 109
**No degeneration**	33 (41%)	20 (25%)	7 (8%)	7 (6%)
**Isolated anterior degeneration**	18 (23%)	22 (28%)	8 (9%)	5 (5%)
**Combined anterior/posterior degeneration**	12 (15%)	23 (29%)	56 (61%)	71 (65%)
**Isolated posterior degeneration**	17 (21%)	15 (19%)	21 (23%)	26 (24%)

*Anterior degeneration: at least moderate disk height loss at L2-S1; posterior degeneration: at least moderate disk height loss at L2-S1

Table 3 presents the prevalence of the four patterns of degeneration by age group, using an alternate definition for anterior degeneration, as the presence of any disk height loss at one or more levels. Isolated posterior degeneration was again noted not to substantially increase or decrease with increasing age group, with 8-12% of individuals demonstrating moderate or severe FJ OA without any DHL.

**Table 3:** Prevalence of Patterns of Spinal Degeneration by Age Group (Definition 2)*

	Age < 45	Age 45-54	Age 55-64	Age 65+
**No degeneration**	16 (20%)	7 (9%)	4 (4%)	4 (4%)
**Isolated anterior degeneration**	35 (44%)	35 (44%)	11 (12%)	8 (7%)
**Combined anterior/posterior degeneration**	22 (28%)	32 (40%)	66 (72%)	87 (80%)
**Isolated posterior degeneration**	7 (9%)	6 (8%)	11 (12%)	10 (9%)

*Anterior degeneration: any disk height loss at L2-S1 (including mild height loss); posterior degeneration: at least moderate disk height loss at L2-S1

In order to account for potential confounding, and to permit investigation of effect modification, the predictor variable of anterior degeneration- as well as the covariates of age, sex, BMI, and smoking- were included in multivariate logistic regression models using the response variable of posterior degeneration. These results are presented in Table 4. Using our primary definition of anterior degeneration (moderate or severe DHL), anterior degeneration (OR 1.93 [95% CI 1.15- 3.25]) was significantly associated with the presence of posterior degeneration (moderate or severe FJ OA). Age in years (OR 1.09 [95% CI 1.06-1.11]), female sex (OR 1.86 [95% CI 1.09- 3.18]), and BMI in units of kg/m^2 ^(OR 1.09 [95% CI 1.03- 1.16]) were also independently and significantly associated with posterior structure degeneration, but smoking was not. When interaction terms were added to the model, there were no significant interactions between moderate DHL and these covariates.

**Table 4:** Multivariate models for the outcome of any posterior degeneration (n = 361)*

	Odds ratio (95% Confidence Interval)
**Model 1 (Disk height loss +Age + Sex + BMI+ Smoking^†^)**	

Disk Height Loss (Moderate/Severe)	1.93 (1.15-3.25)
Age (year)	1.09 (1.06-1.11)
Female sex	1.86 (1.09-3.18)
BMI (kg/m^2^)	1.09 (1.03-1.16)
Smoking^† ^	0.93 (0.42-2.07)

**Model 2 (Disk height loss +Age + Sex + BMI+ Smoking**^†^)	

Disk Height Loss (Any)	1.93 (1.01-3.68)
Age (year)	1.09 (1.06-1.12)
Female sex	1.85 (1.08-3.15)
BMI (kg/m^2^)	1.10 (1.03-1.16)
Smoking^† ^	0.95 (0.43-2.09)

*either isolated posterior degeneration or posterior degeneration in combination with anterior structure degeneration

† Smoking tobacco in the past year

Repeating the multivariate logistic regression, and using our secondary definition of anterior degeneration (any DHL), anterior degeneration remained significantly associated with the presence of posterior degeneration. Age, female sex, and BMI were also independently and significantly associated with posterior structure degeneration, but smoking was not. When interaction terms were added to the model, the interaction of BMI with any DHL was statistically significant (p = 0.02), suggesting a greater effect of increased BMI when any DHL was present.

## Discussion

The current study is consistent with the view that, for a majority of individuals, degeneration begins with the anterior spinal structures. However, a minority of individuals (10-20%, depending on the definition of anterior degeneration used) across the age spectrum exhibits a pattern of isolated posterior degeneration without substantial loss of disk height, occurring most frequently at the L5-S1 and L4-L5 spinal levels. For these individuals, the factors of age, sex, and BMI may explain, at least in part, the development of posterior structure degeneration without concurrent changes in the disk.

Kirkaldy-Willis described spinal degeneration as the result of a complex interaction between the intervertebral disks and facet joints, which begins with precipitating events that could take place in any component of the three-joint complex. He stated clearly, '*In some patients the changes seen during the course of the progressive degenerative process affect mainly the facet joints*' [3]. Our finding that some individuals have changes of facet degeneration without any changes of disk height loss is therefore consistent with the Kirkaldy-Willis view, and furthermore is supported by some earlier studies. The seminal cadaveric study by Lewin reported that FJ OA sometimes occurred in the absence of disk degeneration or vertebral osteophytosis [14]. Eubanks' study of skeletal lumbar spines found that lumbar FJ OA appeared early in the course of aging, often preceding anterior vertebral changes. On the other hand, some cadaveric and imaging studies described above have concluded that disk degeneration always precedes facet degeneration. Taken together, the existing literature suggests that many exceptions exist to the generalization that anterior changes precede posterior changes. These exceptions may be explained by the contributions of increased age, female sex, and higher BMI. However, longitudinal studies in humans are needed to verify whether any of the previously proposed biomechanical, demographic, or anthropometric risk factors for posterior structure degeneration are truly causal. A compelling alternative explanation exists: the predominant influence of heredity in disk degenerative changes has already been shown [31], and heredity may well explain much of the variation seen in facet degenerative changes as well. Future longitudinal studies should ideally allow for examination of genetic factors, and should account for other covariates not considered in this study, including occupational loading, prior physical trauma, lumbosacral alignment, and facet joint orientation and tropism. In particular, our finding that isolated posterior degeneration occurred predominantly at the L5-S1 and L4-L5 levels may warrant more detailed consideration of lumbosacral biomechanical factors. Furthermore, since disk height narrowing is a relatively nonspecific finding with poorly understood determinants, future studies should take into account other parameters of disk degeneration, including quantitative assessments of desiccation, herniation, and annular pathology.

This study has the advantage over prior studies in that we sampled a community-based population, included a large number of subjects, and used well-described and reliable measures for the pathoanatomic features of interest. In addition, our analysis was unique from others examining this question in that we used multivariate techniques. This is especially important because factors such as age are known to have strong positive associations with both DHL and FJ OA, creating the possibility of substantial confounding. We were able to demonstrate associations between key covariates and FJ OA, independent of the main association of interest between DHL and FJ OA.

Some limitations pertain to this study. First, the cross-sectional nature of this study limits firm conclusions about causality. Second, although aspects of quantitative measurement were contained within the categorical scales of DHL and FJ OA used in this study, these scales were not truly quantitative. Some prior studies have had better resolution for detecting changes in disk degeneration when quantitative scales were used [22]. However, we were limited in our ability to use quantitative scales for assessment of FJ OA, because to our knowledge there were no such scales for FJ OA documented in the literature at the time that this study was conducted. Third, the use of CT imaging may be perceived as a limitation of this study. Although CT imaging allows excellent characterization of FJ OA and disk height loss [8], CT is insensitive to early changes of disk degeneration such as disk desiccation, which may be present without substantial loss of disk height. The use of CT could therefore bias against the hypothesis that anterior degeneration precedes posterior degeneration by missing early disk changes. However, prior studies have established that actual DHL is usually present when FJ OA occurs in the setting of disk degeneration [8,12]. Furthermore, transfer of forces to the posterior structures of the lumbar spine through disk narrowing is conceptually a primary mechanism whereby disk changes lead to facet changes [32]. The insensitivity of CT to early changes is therefore unlikely to explain our findings.

## Conclusions

The observed epidemiology of lumbar spinal degeneration in the community-based population is consistent with an ordered sequence beginning in the anterior structures, for a majority of individuals. However, some individuals demonstrate atypical patterns of degeneration, beginning in the posterior joints. Increased age and BMI, and female sex, may be related to posterior degeneration in these individuals. Longitudinal studies are needed to better understand the importance of segmental level biomechanics in degeneration, and ideally should include not only these important covariates, but also examination of genetic factors.

## Competing interests

The authors declare that they have no competing interests, except for the following:

JGJ is a member of the GE Healthcare Advisory Board for Comparative Effectiveness and a consultant for HealthHelp, a Radiology Benefits Management company. AG was previously a scientific advisor for Facet Solutions.

## Authors' contributions

PS was involved with study concept and design, acquisition of data, analysis of data, interpretation of data, and drafting of the manuscript. AM was involved with study design and acquisition of data. DJH was involved with study concept and design, analysis of data, interpretation of data, and manuscript preparation. JGJ was involved with study design and interpretation of data. JR was involved with study concept, design, and interpretation of data. AG was involved with study design and acquisition of data. LL was involved with analysis of data and interpretation of data. JNK was involved with study design, analysis of data, interpretation of data, and manuscript preparation. All authors were involved with critical revision of the manuscript for important intellectual content and approved the final version of the manuscript.

## Pre-publication history

The pre-publication history for this paper can be accessed here:

http://www.biomedcentral.com/1471-2474/12/202/prepub
